# Medical News and Items

**Published:** 1877-02

**Authors:** 


### Medical News and Items.

### THE PREVAILING EPIDEMIC AND THE MEDICAL PROFESSION OF THE CITY.

About 100 medical gentlemen of Chicago assembled at the Grand Pacific Hotel, on the evening of January 25th, in accordance with a request issued by the secretary of the Medical Press Association, at the suggestion of its other officers. The ostensible purpose of the call was the consideration of the invitation made by the Health Commissioner of the city to the medical profession, to furnish him with such counsel as would be most available, in view of the great prevalence of scarlatina and diphtheria, and the resulting mortality in the various wards.

Dr. Hamill, the president of the Association, presided, and Dr. Sawyer acted as secretary. Those who were present wisely concluded not to enter into a discussion of the appropriate treatment of either disease, but to confine the debate to the question of prophylaxis, both by hygienic and medicinal means. As to the latter point great difference of opinion was expressed; not, however, so much on the question of the value of any special remedy as a prophylactic (for it seemed to be the view of almost all present that no such remedy existed), but as to the propriety of issuing a formal statement positively declaring that prophylaxis by internal medication was both valueless and positively injurious. It was finally concluded that the entire question should be submitted to a committee (who were instructed to report at a subsequent meeting) composed of Dr. H. A. Johnson, chairman, and Drs. Ingals, Byford, Etheridge and Jackson.

On the 27th of the month the adjourned meeting, held at the same place, was largely attended; and the committee above named presented their report. The various articles in the latter were separately discussed and argued upon; none meeting with such determined opposition as that relative to the value of preventive medicines. At this time, as at the preceding meeting, the objection was based chiefly, not upon any belief in the value of such medicine, but upon what was considered the impropriety of absolutely discouraging the public from employing remedies which might be supposed to have prophylactic value, even when administered under the direction of a judicious physician. The result finally reached is seen in the subjoined copy of the report. We are of opinion that, taking into consideration the existing prejudices in the minds of many of the laity, and the honest disposition of many medical men to attempt to secure their patients from an imminent danger, the recommendation is a good one.

In all other respects the report of the committee is admirable from every point of view, and was worthy of promulgation to the representative of the Health Department and the public, before whom the report was spread in full by the daily journals of the city. We cannot but think that the gentlemen engaged in this discussion and the preparation of this report, discharged a high public duty by their action, and one which has not failed to heighten public respect for their position and attainments. Subsequently, Dr. Oscar C. DeWolf, a competent and worthy medical gentleman, was nominated by the Mayor of the city as Superintendent of the Department of Health, and this nomination was confirmed by the Common Council. The new incumbent has since proceeded to the discharge of his duties in a manner which promises an improvement in the administration of an important public office, whose action we have had frequent occasion to critizise in these columns.

Let the medical gentlemen of Chicago hereafter nominate the candidates for all positions where the county and city employ physicians, and we shall soon see a desirable divorce between politics and science. Hitherto "politics" has been the greatest enemy of scientific progress. Let us hope that with such nominations in the future, no medical man may be found engaged in the disgraceful and unprofessional task of lobbying for place, and soliciting votes for his appointment to a position of profit.

We feel assured that the assembling of medical men for the discussion of questions of general and public importance, under the auspices of an association whose scope is less limited than that of the medical societies proper, is a step in the right direction; and its substantial fruit has been pointed out. We trust that by the aid of such additional meetings in the future the medical men of Chicago will give some guarantee to the public that they will so regulate the practice of medicine, at least *within their own ranks*, that none but competent men will be found there, to whom the people of the city are invited to entrust their health and their lives.

The following is the report of the committee:

REPORT.

To the Health Department of the City of Chicago: In conformity to the request of your superintendent, a large assembly of physicians convened at the Grand Pacific Hotel on the evening of the 25th inst., to consult on the sanitary condition of the city, and especially as to the most effectual means of checking the ravages of scarlet fever and diphtheria, under which it has for some time suffered. At this time the undersigned were appointed to prepare and present a report on the subject to a subsequent meeting. In fulfillment of the duty thus imposed the subjoined report is made. It is clearly true that the conservation of the public health is one of the most manifest duties of any civilized people, and the subject cannot escape the consideration and care of civil governments. At all times, and especially in times like the present, the health department of the city should be conducted on principles of strict economy, but we think sufficient money could be diverted from our present municipal expenses for the public charities of the county, and that without impairing their usefulness, to give an increased security to the lives and health of those outside of these institutions. Even liberal expenditures would be warranted, if for anything, for the purposes we seek; for aside from the mental and bodily pain that sickness brings to individuals and families, it is the most expensive misfortune that can rest upon a people; and to the poor it is an overwhelming burden.

Disease checks the production of wealth, and necessitates its largely increased consumption in the care of the sick, while the lives that are sacrificed have a substantial value to the state. Viewing the question in this, its lowest aspect, it is clear that a proper regard for our commercial, social and pecuniary interests, demands that a residence in this city should be rendered safe, salubrious and agreeable. Nature has given the surroundings that should make Chicago one of the most healthful cities of the earth, and all that is necessary to the realization of this condition is the intelligent co-operation of its citizens. We think the board of health should be organized with powers adequate to the accomplishment of this purpose.

As to scarlet fever, the more immediate subject of our inquiry, we count it among the most contagious of diseases, and of its fatality the many stricken homes of this and other cities are a painful attestation. The disease when established has a course largely controlled by the laws of its own nature. Most cases, undisturbed in their progress, go on to recovery, while those of aggravated severity are not reprieved from death by the most enlightened and assiduous efforts of both physician and friends. Therefore the public safety should be sought in efforts to prevent this disease from being disseminated. We believe proper methods faithfully carried out, would expel it from the city, and prevent its return in an epidemic form. Toward effecting this the following rules suitable to be observed so far as practicable are submitted:

Scarlatina, scarlet fever, scarlet rash and canker rash are one and the same disease. It is very infectious. A very mild case may give rise by infection to very a severe one. Infection is contained in all discharges from the body during the progress of the disease and recovery, but more especially from the skin during convalescence, and when the cuticle is being shed. The dry particles which are separated from the skin are highly infectious, and retain their infectious nature for an unknown time, unless thoroughly disinfected. They are disseminated through the air, and become attached to articles of furniture, clothing, draperies, wall papers, etc. Thus the disease may readily be conveyed from one person to another by those who are not themselves suffering from it. It is also conveyed by bedding, clothing, furniture, and other articles, and by rooms which having been exposed to infection, have not had their floors, ceilings or walls disinfected, or had the wall paper removed.

Isolate the person affected as much as possible from other inmates of the house. This is most readily effected by at once removing him to an upper room, if circumstances permit. The room selected should be large and airy, and the means of ventilating it, which shall be presently mentioned, at once adopted. No child should be permitted to go to school, or to any public assembly, from an infected house, and communication of such, in play or otherwise, with healthy children should be prevented. When a person has had the disease, he should not be permitted to mix with others until he has perfectly recovered, and has had his clothes thoroughly disinfected; and not even then without the permission of his medical attendant. Nor is it advisable that any one who has had the slightest communication with a person suffering from the disease should go to any church, meeting, public house, fair, or market, etc. Neglect of these precautions is a prolific cause of the spread of this disease.

Attendants on persons suffering from scarlatina should be chosen, if possible, from those who have already had the disease.

There should be no public funerals of any patient who has died of any infectious or contagious disease. Remember that the separation of the sick person from the well is the most certain means of preventing the spread of the disease.

The room must be kept well ventilated under the physician's direction, by means either of a fire (when required) or of an open fire-place and chimney, and of windows opening to the external air. By means of the latter ventilation is most effectually procured, so as to avoid draughts, in the following manner: Raise the lower sash of the window three or four inches, then procure a piece of wood made to fit accurately in the lower opening, and place it there. By these means free outward and inward currents of air, without causing any draughts, are obtained through the vacant space between the two sashes. When the window is merely opened from the upper or lower sash draughts are invariable caused.

Before removing the patient to the room to be occupied, the following preparation ought to be made: All superfluous curtains, carpets, woolen articles, unnecessary clothing, in short, everything likely to retain infection should be at once removed. The patient's bed ought to be so placed as to allow of a free current of air around it, but not so as to place it in a draught.

All sheets, towels, handkerchiefs, etc., which have been used by the patient should be thoroughly disinfected and afterward carefully washed.

In all cases of infectious disease it may be as well that the patient use rags or pieces of old linen, etc., (in lieu of pocket handkerchiefs,) which may afterward be burned, or, better, buried.

When the bed or body linen is soiled, the soiled spots should be sprinkled with some disinfecting powder.

Frequent bathing and the application of oil or glycerine and water, or some such substance under the direction of the physician are advisable. First, they prevent the escape of contagious matters from the body. Second, many physicians believe they are useful.

After the removal of the patient to the room in which he is to remain, the outside of the door and door posts should be completely covered by a sheet kept wetted by some disinfecting fluid, such as the chlorides, sulphates, or sulphates of zinc, lime, soda, iron, etc. Implicit trust, however, should not be placed in the so-called "disinfectant." They are very useful when judiciously employed, but they are by no means certain "preventives of disease."

Rooms which have been occupied by a person suffering from infectious disease should, on the termination of illness, be at once disinfected. To effect this thoroughly, all crevices around windows and doors and the fireplace should be closed by pasting pieces of paper over them. Lumps of sulphur (brimstone), one pound for every thousand cubic feet of space, should then be put into a metal dish, placed by means of tongs over a bucket of water. This being set fire to, the door should be closed, and the room should be allowed to remain without interference for three or four hours. After this time the windows should be thrown open, and when the fumes have disappeared all the wood-work and walls should be thoroughly washed with soft soap and water, to which carbolic acid has been added (one pint of the common liquid to three or four gallons of water), and the paper from the walls stripped off. In white-washed rooms the walls should be scraped and then washed with hot lime.

No reliance ought to be placed upon any medicine given to prevent the disease. Medicines so given may do harm. They should be taken only upon the order of the physician of the family.

WHAT THE AUTHORITIES OUGHT TO DO.

1. Require a report to the health department of every case of contagious or infectious disease.

2. Officially inquire into the origin of disease in each case.

3. Take such measures as will prevent communication between the infected location or house and those not infected, by the placing of placards, and other measures which have been found useful as warnings to the public; and by prohibiting children from infected houses from attending public schools; prohibiting public funerals of persons who have died from infectious or contagious diseases, and by enforcing the disinfection of clothing, furniture, bedding and the rooms used or occupied by persons who have had any contagious or infectious disease.

If additional legislation is required to secure the measures, it should be had without delay.

The committee earnestly recommend the people, the physicians of the city, and the public authorities to adopt and enforce, as far as possible, substantially the measures above recommended. Concerning the utility of these means there is among educated physicians no important difference of opinion. The means proposed and the disinfectants suggested are not expensive.

---

The Pancartes.—Our French exchanges indicate that the medical profession of Paris have been much moved recently in consequence of discussions and correspondence on the subject of the "*pancartes.*" These are a species of card suspended above the head of a hospital patient, indicating his name and other facts relative to his case, of interest to the surgeon. The general superintendent of the Paris Hospitals, a layman, has recently insisted that these cards should state also the religion of the beneficiary, when he or she received the sacrament last, and other items which would be of value only to the priest or sisters of charity. A vigorous public protest against this order has been made by several eminent hospital surgeons who claimed that the sick committed to their professional charge shall not be annoyed by questions intended to ascertain such facts as those required to be stated upon the "*pancarte.*"

There have been rejoinders and sur-rejoinders, but the medical men have been almost unanimous in their condemnation of the edict, while they have been coolly notified by the authorities to attend to their professional duties and leave the religious care of their patients to the clergy and sisterhood.

